# Detection and quantitation of hepatitis B surface antigen (HBsAg) on dried blood spots: a solution for easy access for hepatitis B diagnosis and elimination in remote areas

**DOI:** 10.11604/pamj.2022.42.100.30531

**Published:** 2022-06-07

**Authors:** Anna Julienne Selbé Ndiaye, Halimatou Diop-Ndiaye, Gora Lo, Aminata Dia, Mba El Hadji Bambo Diakhaby, Aissatou Sow, Assane Dieng, Souleymane Mboup, Cheikh Saad Bouh Boye, Coumba Touré Kane

**Affiliations:** 1 Laboratory of Bacteriology Virology, Hospital Center University Aristide Le Dantec, Dakar, Senegal,; 2 Institut de Recherche en Santé de Surveillance Epidémiologique et de Formation, Dakar, Sénégal

**Keywords:** Hepatitis B virus, dried blood spots, HBsAg quantification

## Abstract

Hepatitis B virus (HBV) is generally endemic in resource-limited countries, which are characterized by a deficit of technical facilities that could delay diagnosis and treatment. To facilitate the accessibility to diagnostic and connection to treatment, evaluation, and promotion of alternatives and/or simplified strategies and inexpensive tools such as dried blood specimens need to be investigated and implemented. This study aimed to evaluate dried blood spots (DBS) for the detection and quantification of HBsAg. This study included 100 DBS from subjects tested positive for HBsAg, and 50 DBSs from subjects tested negative for HBsAg by the automate Architect i1000sr (Abbott Diagnostics, Ireland). Hepatitis B surface antigen detection was performed with determine HBsAg Alere® tests (Alere International Limited, Ireland) and Architect® HBsAg Qualitative II Assays (Abbott, Diagnostics, Ireland) after 15 and 30 days (D15, D30). For HBsAg-positive subjects, the quantification of HBsAg was performed at day zero (D0) from plasma and at D15 and D30 from the DBSs. At D15, the sensitivity and specificity were 96% and 100% for the Determine® tests and 100% and 100% for the Architect® tests, respectively. At D30, the sensitivity and specificity were 96% and 100% for the Determine® tests and 100% and 100% for the Architect® tests, respectively. For HBsAg quantification, the agreement rates were 96%, 96% and 100% between D0-D15, D0-D30 and D15-D30, respectively. This work showed that DBSs can be very useful for HBsAg detection and quantification and therefore in the management of HBV infection in resource-limited settings.

## Introduction

With a total of 257 million hepatitis B virus (HBV) chronic carriers, hepatitis B infection remains a major public health problem, especially in endemic areas [1]. In 2015, 887,000 people died from complications related to this infection, in particular cirrhosis and hepatocellular carcinoma [1], making HBV the third leading cause of cancer death after tobacco [2]. Sub-Saharan Africa is a highly endemic area for HBV, with a prevalence of HBsAg carriage exceeding 8% [1,3,4]. In Senegal, 85% of the general population has been in contact with at least one viral marker, and 11% are chronic carriers of HBsAg [5-7]. To prevent complications, hepatitis B early diagnosis is essential for a precocity of curative management that will help to block viral replication and control the progression of the disease. HBsAg screening is necessary to establish the patient's status, and to initiate the detection of other markers including HBV deoxyribonucleic acid (DNA) quantification which is essential for virologic monitoring of infected patients [1]. Likewise, the quantification of HBsAg allows the evaluation of the response to treatment and its duration, and the loss of HBsAg [8-10].

Automated platforms for diagnosis like Architect Analyzer (Abbott Diagnostics, Ireland) are widely used in developed regions and have been validated for both plasma or serum [5,11,12]. However, in limited-resource settings, the deficit of technical platforms prevents access to these automated diagnosis techniques especially, in remote regions. Therefore, as for HIV testing, using DBSs could improve the access to hepatitis B diagnosis for populations in remote and rural areas [12-15]. This large access could be emphasized by the combination of DBS and rapid diagnostic tests. This study aimed to assess the performance of DBSs in the detection and quantification of HBsAg.

## Methods

**Patients and samples:** this prospective study was performed at the Bacteriology-Virology Laboratory of the University Hospital Center Le Dantec, Dakar, Senegal. Whole blood samples were collected in ethylenediamin tetra-acetic acid (EDTA) tubes from patients after informed consent.

**Specimen preparation:** from each blood sample collected from patient, an aliquot was centrifuged to obtain plasma. Testing for HBsAg was first performed on 125 μl of plasma with the automate Architect HBsAg Qualitative II assay (Abbott Diagnostics, Ireland), with a positive result if s/co value (Relative Light Units (RLUs) value of the sample/ RLUs value of the threshold value) is greater than 1. Therefore, a total of 100 positive samples for HBsAg and 50 negative samples were retained to prepare the DBSs. Fifty microliters of venous blood were then deposited on each of 5 circles of Whatman 903™ filter paper (GE HealthCare, Freiburg, Germany). After drying, the DBS samples were stored at room temperature (22-25°C) in the presence of desiccants and humidity indicators until detection and quantification tests that were completed at 15 days (D15) and 30 days (D30).

**Detection of HBsAg from DBSs:** at D15 and D30, 3 spots measuring 6 mm each were cut from each DBS card and then eluted in 600 μL of phosphate buffered saline (PBS) buffer for 2 hours at room temperature. The qualitative detection of HBsAg in the eluates was performed using Architect HBsAg Qualitative II and Determine® HBsAg (Alere International Limited, Ireland) kits according to the manufacturer´s instructions.

**Quantification of HBsAg from plasma and DBS:** quantification of HBsAg was performed at D0 from 150 μl of plasma for each of the 100 samples positive for HBsAg and from the DBS samples at D15 and D30. Given the dilution of the blood by the elution of DBS and the hematocrit level, all the quantification values obtained at D15 and D30 were corrected by a conversion factor of 35.58. The cutoff point for the positive quantification of HBsAg was 0.05 IU/ml [16].

**Statistical analysis:** the results obtained for the detection and quantification of HBsAg on plasma and the DBSs (D15 and D30) were entered into Microsoft Excel and analyzed with Epi Info 3.5.1. (CDC, Atlanta, GA) with a 95% confidence interval (95% CI). For HBsAg detection, the results from Determine® HBsAg and Architect HBsAg Qualitative II tests on the DBSs (D15 and D30) were compared with those obtained by the Architect HBsAg Qualitative II assays on plasma by calculating the sensitivity (Se), specificity (Sp), positive predictive value (PPV), and negative predictive value (NPV). For HBsAg quantification, the results obtained on the DBSs (D15 and D30) and plasma were converted into logarithmic (log) values and were compared to the values obtained with plasma. The log difference was calculated, and any value greater than 1 log was considered significant. In addition, correlation and concordance were determined by regression line calculation and Bland-Altman plots with MethVal software.

## Results

**Qualitative detection of HBsAg in plasma with the Architect HBsAg Qualitative II test:** HBsAg-positive plasma samples (n=100) exhibited optical densities (s/co) ranging from 124.7 to 6626.75 with a median value of 3626.84, and 99% had an Optical Density (OD) > 1000 s/co. All negative plasma samples (n=50) presented a s/co ratio less than 1.

**Qualitative detection of HBsAg on DBS with the Architect HBsAg Qualitative II test:** the eluates of the 150 DBS were all tested at D15 and D30 with the Architect® HBsAg Qualitative II test for HBsAg. A 100% agreement was noted between plasma and DBS at both D15 and D30 (Table 1), with sensitivity, specificity, positive predictive value, and negative predictive value of 100%, 100%, 100% and 100%, respectively. At D15, the s/co values of the positive DBS samples varied from 2.04 to 5795.02 s/co with a median value of 2396.97, and at D30, the DBS eluates that tested positive had optical densities varying from 1.27 to 5532.38 s/co with a median value of 1750.96.

**Table 1:** results of the architect qualitative II test in the qualitative detection of HBsAg from DBSs at D15 and D30

Plasma		DBS D15		DBS D30	
		Positive	Negative	Positive	Negative
	Positive	100	0	100	0
	Negative	0	50	0	50

**Qualitative detection of HBsAg on DBSs with the Determine® HBsAg test:** among the 150 DBSs tested on Determine® HBsAg, 4 positive plasma samples were discordant, testing negative on their corresponding DBSs (D15 and D30), resulting in an overall agreement of 97.3% (Table 2). Therefore, the Determine® HBsAg test with the DBSs presented a sensitivity, specificity, positive predictive value, and negative predictive value of 96%, 100%, 100% and 92.5%, respectively, at D15 and D30. The 4 DBSs with discordant results between the Architect HBsAg Qualitative II and Determine® HBsAg tests had optical density s/co values less than 200 on the Architect tests at D15 and D30 (Table 3).

**Table 2:** results of the determine® HBsAg test in the qualitative detection of HBsAg from DBSs at D15 and D30

Plasma		DBS (dried blood spot) D15		DBS D30	
		Positive	Negative	Positive	Negative
	Positive	96	4	96	4
	Negative	0	50	0	50

**Table 3:** discordant results between the Architect HBsAg qualitative and determine HBsAg assays

	Architect® HBsAg qualitative II				Determine® HBsAg	
	OD (s/co) plasma	OD (s/co) D15	OD (s/co) D30	Interpretation	D15	D30
HB 434	2805.83	184.66	174.69	Positive	Negative	Negative
HB 582	4246.66	94.99	94.28	Positive	Negative	Negative
HB 764	4040.52	72.58	45.18	Positive	Negative	Negative
HB 799	124.77	86.1	74.71	Positive	Negative	Negative

**Quantification of HBsAg in plasma:** the quantification of HBsAg from the 100 positive plasma samples showed concentrations ranging from 3.67 IU/ml (0.56 log IU/ml) to 50,208.61 IU/ml (4.70 log IU/ml), and the median value was 6658.38 IU/ml (3.82 log IU/ml).

**Figure 1:**
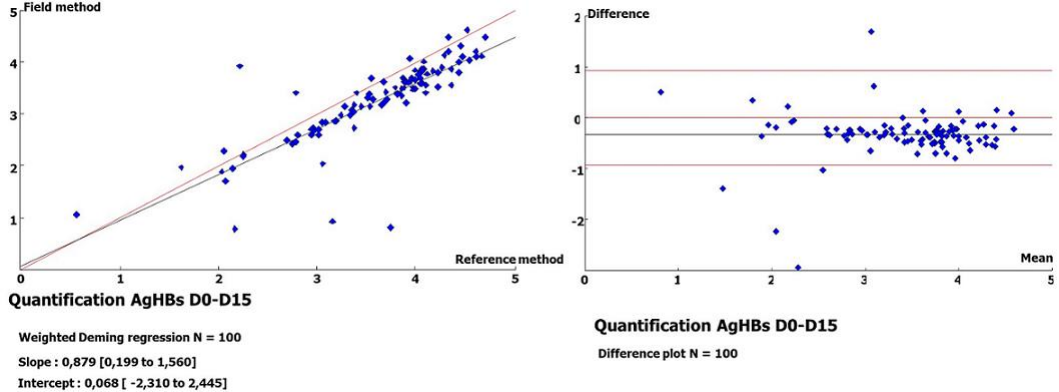
regression line and Bland-Altman plots for quantitated HBsAg from plasma at day 0 versus dried blood spots at day 15

**Quantification of HBsAg on DBS on D15 versus plasma:** at D15, the HBsAg concentrations varied between 6.04 IU/ml (0.78 log IU/ml) and 40,675.41 IU/ml (4.60 log IU/ml) with a median value of 2739.30 IU/ml (3.43 log IU/ml). The comparison between plasma and DBS at D15 showed a concordance rate of 96% (n= 96) with a log difference less than 1 (not significant), and only 4% (n=4) presented a log difference ≥1. Moreover, the comparison of the quantification of HBsAg between D0 in plasma and D15 for the DBS showed a good correlation with r=0.82 and a bias (mean of the log differences) of -0.332 (- 0.424 to - 0.230), with 5 samples outside the 95% confidence interval (Figure 1).

**Figure 2:**
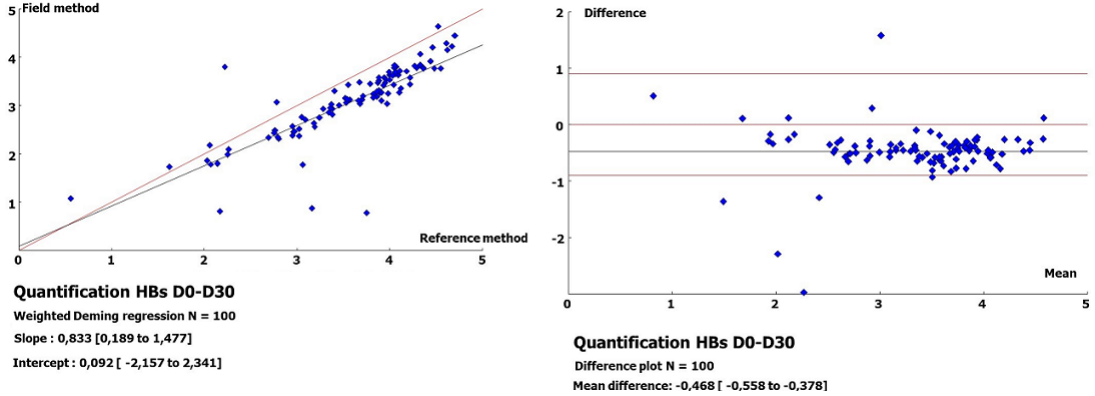
regression line and Bland-Altman plot for quantitated HBsAg from plasma at day 0 versus dried blood spots at day 30

**Quantification of HBsAg on DBS at D30 versus plasma:** at D30, the HBsAg concentrations varied between 6.04 IU/ml (0.78 log IU/ml) and 43,093.42 IU/ml (4.63 log IU/ml) with a median value of 1798.03 IU/ml (3.25 log IU/ml). Less than 1 log difference was noted between D0 and D30 for 96% (n=96) of the samples versus 4% with a log difference ≥1. By comparing the quantification of HBsAg in plasma at D0 and at D30 for the DBS, regression analysis showed a good correlation (r=0.83), and the Bland-Altman plot indicated a bias of -0.408 (-0.558 to -0.378), with 5% of the samples falling outside the 95% confidence interval (Figure 2).

**Figure 3:**
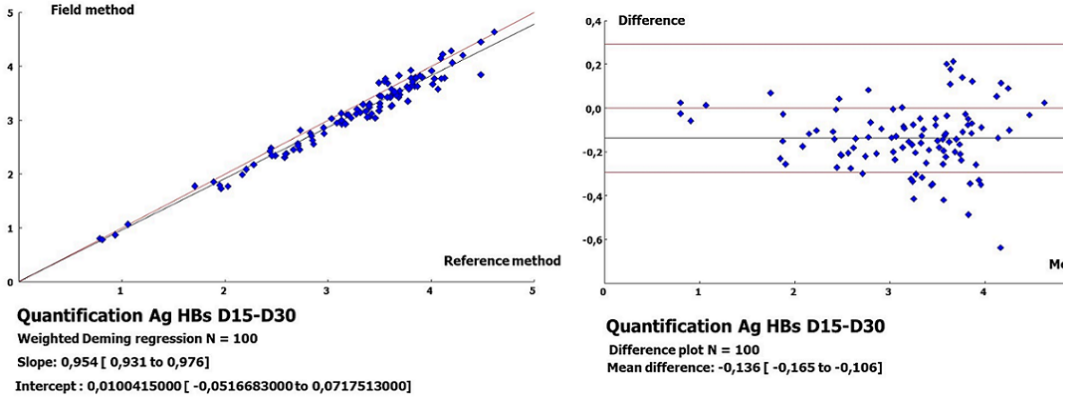
regression line and Bland-Altman plots for quantitated HBsAg from dried blood spots at day 15 versus day 30

**Quantification of HBsAg from DBSs on D15 versus DBSs on D30:** the HBsAg quantification values between D15 and D30 showed no variation since all samples had a log difference <1. In addition, comparison of the quantified HBsAg from DBSs between D15 and D30 showed a strong correlation, with a coefficient of r=0.98 and a bias of -0.136 (-0.165 to -0.106), with 10 samples falling below the lower limit of the 95% confidence interval (Figure 3).

## Discussion

Since May 2010, hepatitis has been considered the fourth-most prioritized global public health concern by the World Health Organization (WHO) after HIV/AIDS infection, malaria, and tuberculosis [17-19]. The current strategy for fighting against hepatitis aims to reduce the number of new infections by 90% and the number of deaths from viral hepatitis by 65% by 2030 relative to 2016 [17-19]. In sub-Saharan Africa, where hepatitis B is endemic, there is generally no national systematic screening strategy for the disease [20]. The screening, diagnosis and follow-up of patients are made difficult by the lack of simple and reliable diagnostic tools in peripheral laboratories [17,18,20]. According to the WHO, the priority is to move towards rapid and feasible diagnostic tests at the point of care in remote areas [17,18]. The combination of the use of DBSs and rapid diagnostic tests could facilitate the storage and transport of specimens to reference laboratories and improve accessibility to the diagnosis of this infection.

This study aimed to evaluate the performance of DBSs with two techniques using plasma as a reference sample. In the qualitative detection of HBsAg, the results indicated a good performance from the Determine® HBsAg and Architect® HBsAg tests with DBSs, with respective sensitivities of 96% and 100% at D15 and 96% and 100% at D30 and an excellent specificity of 100%. Several studies have also evaluated DBSs by immunochromatography [21]. Enzyme-linked immunosorbent assay (ELISA) [14,22,23] and chemiluminescence [12,13,24,25], with similar sensitivities and specificities ranging from 96% to 100% in the detection of HBsAg. Furthermore, good stability of HBsAg from DBSs at room temperature (25-30°C) was noted regardless of the test used (Architect or Determine). Likewise, low variability has been noted in the detection of HBsAg from DBS except when stored at 42°C [12,26]. On the other hand, a loss of HBsAg from DBSs was noted with different storage temperatures except those of -20°C and -70°C [27]. This difference suggests that it would be necessary to determine the optimum storage temperatures for DBSs.

In the quantification of HBsAg from plasma, the results obtained varied between 3.67 and 50208.6 IU/ml (0.56 - 4.70 log IU/ml). In addition, the comparison between the quantification values of HBsAg from plasma and DBSs showed good correlations (r=0.82 and 0.83), with biases of -0.332 and -0.408, respectively, on D0-D15 and D0-D30. A better correlation (r=0.98) with a bias of -0.17 was found in the quantification of HBsAg on the Architect platform from DBSs [24]. This improved performance over that of our study could be linked to the elution of the entire spot (versus 3/4 of the spot) in a volume of 450 μl of PBS buffer (versus 600 μl) under continuous stirring, followed by a centrifugation step at 1000 g. This assessment is supported by the low correlation (r=0.432) obtained on the same platform, which is probably linked to the elution of a quarter (1/4) of the spot and to the solvent used (500 μl of Milli-Q water) [28]. Apart from the elution of the entire spot in 1000 μl of PBS, other studies highlight the importance of the volume of the blood spot (75-100 μl/spot) in the detection of HBsAg with a similar performance [12,13]. In fact, better standardization is needed to obtain reproducible results. Nevertheless, in our study, a strong correlation (r=0.98) with a bias of -0.136 was noted in the comparison of the quantification values of HBsAg from DBS between D15 and D30, thus demonstrating its weak variability over time.

## Conclusion

This study showed that the diagnosis of hepatitis B from DBS specimens is feasible. Therefore, DBSs could be an alternative tool that could be implemented and reach populations in remote areas where there are difficulties in transporting fresh blood samples to referral centers. Optimization studies should be continued with the goal of validating this tool, which could help systematize HBsAg screening, improve the care and follow-up of patients infected with HBV, and vaccinate patients with a negative HBV serology. Finally, this study underlines the importance of this alternative method, which can lead to recommendations for decision-makers and providers for a reorientation of national strategies in the diagnosis and management of HBV in Senegal and other highly endemic, resource-limited countries and may serve as a key element that contribute to reaching the 2030 goals.

### What is known about this topic

- Access to hepatitis B diagnosis is a difficulty in remote areas where technical facilities are not available.

### What this study adds

- Feasibility of hepatitis B diagnosis from DBS specimens;
- Possibility for remote areas having difficulties in transporting fresh blood samples to have DBS as an alternative tool.
